# Snowshoe hares display limited phenotypic plasticity to mismatch in seasonal camouflage

**DOI:** 10.1098/rspb.2014.0029

**Published:** 2014-05-07

**Authors:** Marketa Zimova, L. Scott Mills, Paul M. Lukacs, Michael S. Mitchell

**Affiliations:** 1 Wildlife Biology Program, Department of Ecosystem and Conservation Sciences, University of Montana, Missoula, MT 59812, USA; 2 US Geological Survey, Montana Cooperative Wildlife Research Unit, University of Montana, Missoula, MT 59812, USA

**Keywords:** camouflage mismatch, crypsis, phenology, phenotypic plasticity, climate change, snowshoe hare

## Abstract

As duration of snow cover decreases owing to climate change, species undergoing seasonal colour moults can become colour mismatched with their background. The immediate adaptive solution to this mismatch is phenotypic plasticity, either in phenology of seasonal colour moults or in behaviours that reduce mismatch or its consequences. We observed nearly 200 snowshoe hares across a wide range of snow conditions and two study sites in Montana, USA, and found minimal plasticity in response to mismatch between coat colour and background. We found that moult phenology varied between study sites, likely due to differences in photoperiod and climate, but was largely fixed within study sites with only minimal plasticity to snow conditions during the spring white-to-brown moult. We also found no evidence that hares modify their behaviour in response to colour mismatch. Hiding and fleeing behaviours and resting spot preference of hares were more affected by variables related to season, site and concealment by vegetation, than by colour mismatch. We conclude that plasticity in moult phenology and behaviours in snowshoe hares is insufficient for adaptation to camouflage mismatch, suggesting that any future adaptation to climate change will require natural selection on moult phenology or behaviour.

## 1. Introduction

Phenological shifts in plant and animal populations have been linked widely to climate change [1,2]. Pressing questions of interest include how these phenological shifts link mechanistically to climate variables and whether the observed shifts are adaptive. Although evolution by natural selection is a possible means of adaptation, the most immediate adaptive solution to the rapid pace of climate change is phenotypic plasticity [3], the range of phenotypes expressed by a genotype in different environmental conditions. In some cases, plasticity in circannual behaviour and other traits has explained most of the observed change in phenology [4,5] and has been shown to maintain or increase fitness [6]. For instance, plasticity in phenology of egg laying in a population of great tits (*Parus major*) in the United Kingdom was adaptive in minimizing phenological mismatch with food sources, thereby maintaining population growth [7].

Snowshoe hares (*Lepus americanus*), and at least nine other mammal species globally, undergo seasonal moults to a white or brown coat to match the presence or absence of snow. Background matching is a crypsis strategy that reduces risk of detection by predators [8,9]. In recent decades, persistence of snow cover in the Northern Hemisphere has decreased owing to increased air temperatures and more precipitation falling as rain instead of snow [10–12]. In a recent study of the mistiming between presence of ground snow cover and seasonal colour moults in snowshoe hares, strong background mismatch was documented during spring and autumn, and predicted to greatly increase in the future [13]. White animals on non-snowy backgrounds are extremely conspicuous and would appear to be easier to detect by a predator that hunts visually. Both individual behaviours and population dynamics of hares are overwhelmingly shaped by predation, which can comprise 85–100% of mortality [14].

Snowshoe hares might be able to minimize fitness costs of seasonal mismatch in camouflage through plasticity in the phenology of coat colour moults. Timing of moult in autumn and spring is presumably initiated by photoperiod, but temperature and possibly presence of snow may affect the rate of the change [15–17]. Mills *et al*. [13] showed average timing of the autumn and spring moult in snowshoe hares in Montana to be fixed across years with disparate snow cover, with some plasticity in the rate of spring moult, once the white-to-brown transition had been initiated.

Separate from or in combination with phenological shifts, hares might be able to modify their behaviour to minimize the mismatch of coat colour or its potential costs. Snowshoe hares rely strongly on their crypsis to avoid predation, with minimal attempts at hiding in vegetation resulting in low concealment. In contrast to other lagomorphs in the region that stay brown during the winter (i.e. mountain cottontails (*Sylvilagus nuttallii*) or pikas (*Ochotona princeps*)), snowshoe hares do not build burrows for escape underground. Rather, during the day, hares sit completely still at a resting spot and do not flee until immediate danger arises. Intuitively, this strategy is maladaptive when selection of a resting spot results in mismatch between coat colour and background. Steen *et al*. [18] observed that willow ptarmigans (*Lagopus lagopus*) moulting from white to brown plumage during snow melt fed in areas that matched their coloration, even though areas selected for optimal crypsis often offered less nutritious food. Similarly, hares could be resting during the day at spots where background colour is similar to their coat colour, and thus reduces colour contrast. Whether hares are able to recognize their coat colour and choose resting spots that match it is not known. Alternatively, mismatched hares might achieve effective camouflage by selecting resting spots associated with cover provided by dense understory, trees or rocks, as suggested by [19]. Finally, because hares rely on both crypsis and flight, mismatched hares may increase the distance at which they flee when approached by a predator [20]. Crypsis in prey species decreases the risk perceived by an animal and consequently can decrease flight initiation distance (FID) [21,22]. For example, round-tailed horned lizards (*Phrynosoma modestum*), whose colouring resembles small stones, displayed shorter FIDs on rocky substrates than on uniform sand, likely as a response to higher crypsis among rocks [23]. Similarly, hares mismatched to their background might perceive higher predation risk and flee sooner (longer FID).

Here, we provide an evaluation of the adaptive potential of snowshoe hares to minimize negative effects of colour mismatch through phenotypic plasticity in moult phenology and behaviour. Mills *et al*. [13] found little plasticity in seasonal coat colour change at a single study site (Seeley Lake, MT, USA). We extend that investigation to a second site which differed considerably in climate and photoperiod and compare moult phenologies at both sites. Next, we analyse at both sites variables influencing moult phenology and explore reaction norms in moult phenology. Lastly, we examine whether anti-predatory behaviours are being modified in response to colour mismatch and consequently evaluate whether behavioural plasticity may ameliorate negative effects of colour mismatch.

## 2. Material and methods

### (a) Study area

We conducted our research at two sites in western Montana, USA, separated by approximately 330 km: the Seeley Lake study site (used in [13]) in the Lolo National Forest (Morrel Creek drainage) and the Gardiner study site in the Gallatin National Forest (Bear Creek drainage). The Gardiner study site is about twice as high in elevation (2400–2700 m.a.s.l.) as the Seeley Lake study site (1300–1450 m.a.s.l.). This elevation difference leads to cooler temperature and longer duration of snow cover in the Gardiner study site; snowpack persists at the Gardiner site from late October until May [24], compared to December to April at the Seeley Lake site [13]. The Seeley Lake site (Lat. = 47.23°, Long. = −113.43°) is 240 km further north than the Gardiner site (Lat. = 45.08°, Long. = −110.57°).

Both sites are temperate boreal coniferous forest on US Forest Service lands with little to no permanent human habitation, and logging being the primary land use. Common predators of hares in these sites include Canada lynx (*Lynx canadensis*), bobcat (*Lynx rufus*), coyote (*Canis latrans*), red fox (*Vulpes vulpes*), American marten (*Martes americana*), great horned owl (*Bubo virginianus*) and northern goshawk (*Accipiter gentilis*).

### (b) Capture and handling

We captured hares at each study site throughout the year in live traps (Tomahawk Live Trap Company, Tomahawk, WI, USA), then ear-tagged, weighed and sexed each individual; hares weighing over 900 g (=199 individuals) were fitted with VHF radiocollars (weight less than or equal to 40 g, Wildlife Materials, Murphysboro, IL, USA; [25,26]).

### (c) Moult phenology

At the Gardiner site, we applied the methods used by Mills *et al*. [13] at the Seeley Lake site, of visually locating hares weekly using radiotelemetry to quantify coat colour phenology and colour contrast between hares and their background. We monitored 51 hares (32 different hares in 2011, and 31 in 2012) at the Gardiner study site and 148 hares (43 different hares in 2010, 63 in 2011 and 58 in 2012) at the Seeley Lake study site. The percentage of white coat colour (% whiteness) and the percentage of ground snow cover within 1- and 10-m radii circles centred at each hare's resting spot (% snow cover) were visually estimated in 20% increments with a standardized protocol of observation and photographs to control for light conditions and distance. All final percentages were visually estimated by a single observer using primarily the photographs, and secondarily the field visual estimates when the quality of the photograph was insufficient, did not show the whole hare's body, or the photograph was absent. We classified animals that just initiated or nearly completed the moult as 5 or 95% white. We measured colour contrast as the difference between per cent whiteness of the hare and the per cent snow cover. It is not known at which scale crypsis may be perceived by either prey or predators; thus, we measured colour contrast within the 1- and 10-m radii circles. We chose these radii because we felt that they were reasonable approximations of the finest and coarsest scales at which crypsis could be effective (i.e. crypsis would be completely ineffective at less than 1 m and completely effective at more than 10 m). A positive contrast indicated a white hare on a non-snowy background, whereas a negative value indicated a brown hare on a snowy background. We considered a hare mismatched when the absolute difference (here referred to as contrast) between its coat colour and background was at least 60% (see [13] for consideration of other thresholds), as at this threshold hares began to clearly stand out against their surroundings.

### (d) Anti-predatory behaviours

We evaluated behaviour of each located hare at both sites. For hares stationary at a resting spot, we visually estimated concealment as the percentage of the hare's body hidden by vegetation at four levels (1 = 0–25% of body concealed to 4 = 75–100% concealed), from the direction from which the hare was initially sighted and from a low angle (approx. 1 m above ground, mimicking the view of common mammalian carnivores). To estimate FID, observers approached a hare at a consistent walking pace (approximately 0.5 m s^−1^) until the hare fled or the observer was within 3 m of the hare. We used a digital laser rangefinder (Leupold, Beaverton, OR, USA) to estimate FID to the nearest metre. We used a minimum approach distance of 3 m to minimize disturbance to the hare; hares that did not flush at the 3-m distance were recorded as ‘no flush’ and were not disturbed further. The maximum distance at which we were able to estimate distance reliably in the forest was 20 m.

Finally, we tested whether hares randomly chose spots to rest with respect to minimizing colour contrast or snow presence in their immediate vicinity. We estimated percentage of snow cover (20% increments) at eight, non-overlapping subsections of the 10-m radius circle around each hare by photographing the ground from where the hare rested at each cardinal and inter-cardinal direction to create eight ‘pie slices’. Snow cover and colour contrast at these eight ‘available’ spots were compared to that in the 1-m radius immediately surrounding the hare's resting spot; we excluded from analysis resting spots where all subsections and the resting spot were entirely snow-covered or snow-free, as these cases provided no information on whether the chosen resting spot differed from the surroundings. Our final sample sizes for this analysis yielded a total of 251 observations from 77 individual hares.

### (e) Statistical analysis

#### (i) Moult phenology

We used a mixed effects change point analysis to estimate the population mean initiation and completion dates of coat colour change phenology in the Gardiner site (as previously done for the Seeley Lake site [13]), and to test for the effects of snow, temperature and sex on moult phenology at both sites. We assessed temperature as the rate of seasonal cooling in the autumn and warming in the spring; we calculated degree days for each day as the cumulative sum of mean temperature below 0°C in the autumn (September–December) and above 0°C in the spring (March–June).

We were able to document individual moult phenology over more than 1 autumn or spring moult for none of the hares at the Seeley Lake site and only seven individuals in the Gardiner site due to the high mortality rates typical of snowshoe hares [25,27] and due to incomplete detection. We plotted coat colour observations over time of those seven individuals for visual assessment of the reaction norm (range of phenotypes produced by a particular genotype in different environmental conditions) in the moult phenology.

#### (ii) Concealment

To test whether hares adjusted hiding behaviour to increase concealment in response to colour contrast, we fitted linear mixed effects models in software R v. 2.15.2 [28] using the package lme4 [29]. We included the identity of individual hares as a random effect to control for variation among individuals. We included the fixed effects of coat colour (per cent whiteness), snow cover around hares (at 1- and 10-m radius), coat colour contrast (at 1- and 10-m radius; ranging from −100 to 100), coat colour mismatch (at 1- and 10-m radius), site, sex and season. Coat colour mismatch was a categorical variable distinguishing between positive (white hare on brown background: contrast ≥ 60), negative (brown hare on white background: contrast ≤ −60) and no mismatch (−60 < contrast < 60). Further, to examine whether the two different types of contrast and mismatch (positive and negative) had equivalent effects, we established an absolute contrast covariate (ranging from 0 to 100) and a binary categorical covariate for absolute mismatch (mismatch: contrast ≤ −60 and contrast ≥ 60 versus no mismatch: −60 < contrast < 60). In addition to linear terms, we used quadratic terms to allow for the possibility of a curvilinear response of concealment to contrast. We differentiated seasons separately for the two sites based on local climate (Seeley Lake: winter (December–March), spring (April–May), summer (June–August), autumn (September–November); Gardiner: winter (November–April), spring (May–June), summer (July–August), autumn (September–October)).

Because hare whiteness and snow cover at both the 1- and 10-m radius around hares were highly correlated for most of the year (*r* ≥ 0.8), we considered each separately in model construction. Whiteness and contrast as well as snow cover and contrast were not highly correlated (*r* ≤ 0.1 and *r* ≥ −0.5, respectively) and so were both present in some models. To test for habituation in hiding behaviour to human observers, we ran a univariate linear mixed model, with concealment as a function of number of location attempts per hare (including unsuccessful sightings) and individual hares coded as random effects to control for variation among individuals.

We selected a set of best models (within 2 ΔAIC*_c_*) fitted with maximum likelihood using AIC*_c_* criterion [30]. The precision of model parameters was based on a sample (100 000 iterations) from the posterior distribution of the fixed effects parameters using a Markov chain Monte Carlo approach (function mcmcsamp) to determine whether the 95% highest posterior density (HPD) intervals included zero.

#### (iii) Flight initiation distance

We used Cox proportional hazards regression [31] to test whether FID increased with colour mismatch. Our data were a form of time-to-event data, with flight the event of interest and observations with no flight response at the maximum approaching distance of 3 m classified as right-censored data. We fitted the models using the package survival [32] in R. We included the same covariates and potential correlations among them as in the previous analysis: whiteness, snow cover around hares, colour contrast (regular and absolute), colour mismatch (regular and absolute), site, sex, season and concealment. We also tested for potential habituation in FID behaviour as in the previous analysis. The proportional hazard assumption was tested using score test and scatterplots of scaled Schoenfeld residuals. We selected a set of best models (within 2 ΔAIC*_c_*) fitted with maximum likelihood using AIC*_c_* criterion.

#### (iv) Resting spots

To test whether hares chose resting spots randomly with respect to colour contrast and snow cover, we fitted separate mixed effects models with fixed effects of colour contrast and snow cover. The models were fitted with binomial error distribution and a logit link function in R using the package lme4 [29]. The binomial dependent variable coded as one for the immediate resting spot (1 m) and zero for the eight other available ‘pie slices’ within the 10-m radius of the hare. The identity of individual hares and the date of when each hare was located were included as random effects to control for variation among individuals and to specify a nested design of the nine spots available to a hare at each location. We compared importance of the tested predictors using the statistical significance of the fixed effects slopes and the models’ AIC*_c_*.

## 3. Results

### (a) Phenology

**Figure 1.**
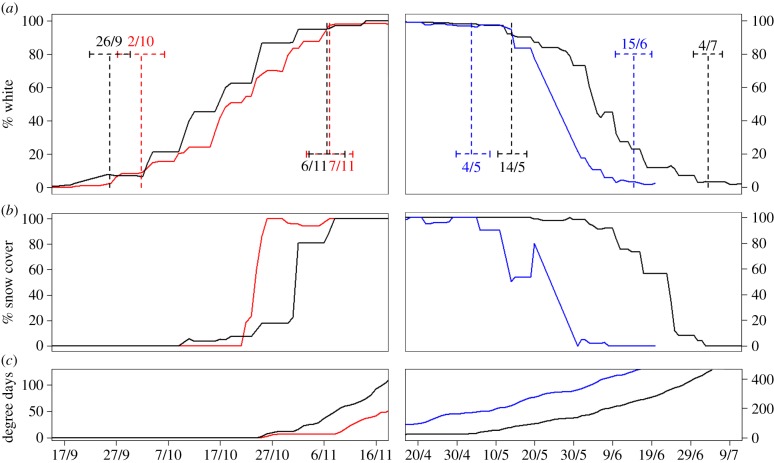
Coat colour phenology, snow cover and degree days at the Gardiner study site, MT (17/9/2010 to 9/7/2012), with autumn seasons on the left and spring seasons on the right*.* (*a*) Weekly average of observed coat colour of 51 hares (2010 (red), 2011 (black) and 2012 (blue)). Dotted lines show the results of Bayesian change point analyses, giving the 95% credible intervals for the mean dates of initiation and completion of the colour moult for each season each year. (*b*) Weekly average of observed snow cover in a 10-m radius around each hare. (*c*) Degree days as a measure of cooling trend in the autumn and warming trend in the spring.

The colour moult phenology analysis at the Gardiner site in two years that differed strongly in amount of snowpack indicated that drivers of this circannual trait, and plasticity across different snow years, were similar to our previous findings at the Seeley Lake study site [13]. The autumn moult for hares in the Gardiner site was fixed across 2011 and 2012 both for initiation date and rate of change (overlapping confidence intervals among initiation and completion dates; figure 1). For the spring moult, we detected plasticity in the rate of the white-to-brown moult. The completion date of the spring moult occurred 19 days later in 2011, consistent with the month longer snow duration in that year (figure 1).

Analysis of the spring initiation date in the Gardiner site was compromised by a small sample size in spring 2012. Specifically, only three radiocollared hares were alive between 4 May and 30 May, as 16 out of 19 hares were depredated in April and early May, and new hares were not collared until early June (see the electronic supplementary material, S1). Thus, the model likely underestimated the initiation date of the spring 2012 moult, leading to the 95% credible intervals of initiation dates between the two years being separated by one day in timing (figure 1).

The limited sample size in spring 2012 also restricted our analysis of the effects of snow cover, temperature and sex on the rate of the spring moult in the Gardiner site by biasing model results. Thus, we combined the Gardiner site data and the Seeley Lake site data from springs 2010 to 2012 to test for the effects of the covariates on the spring moult rate using a larger sample size. Snow cover was negatively related to the rate of change, but the magnitude of the effect was small. A change from 100 to 0% snow shifted the average completion date of the spring moult by only three days (*β*_Snow_ = 0.054, s.d. = 0.015). Temperature also had an effect on the rate of the moult, but the coat colour phenology model with temperature (degree days) as a covariate predicted that the span from 0 to 23.1°C (the highest daily average temperature during the spring moult period) explained only a one-day modification of the completion date of the spring moult (*β*_Temp_ = 0.15, s.d. = 0.016). Lastly, when testing for the effects of sex on spring moult rate at the two sites, the sex-skewed spring data from the Gardiner site in 2012 positively biased the estimate. In spring 2012, five out of the seven hares observed to change to 5 or 0% white were females, which indicated that females completed the spring moult 14 days earlier than males. By contrast, omitting the Gardiner spring 2012 data resulted in a minimal influence of sex on the rate of the spring moult, with females completing the spring moult on average two days earlier than males (*β*_Sex_ = −4.43, s.d. = 5.27).

We observed a large difference in the timing of the coat colour moult phenology between our two study sites. For each year, hares at the Gardiner site initiated autumn moults by one to two weeks earlier in the autumn and by a month later in the spring, corresponding to cooler temperatures and longer lasting snowpack in the Gardiner site. However, the duration of the colour moults was very similar across sites with autumn and spring moults lasting on average 39.9 days (s.d. = 3.22) and 41.9 days (s.d. = 7.00), respectively.

**Figure 2.**
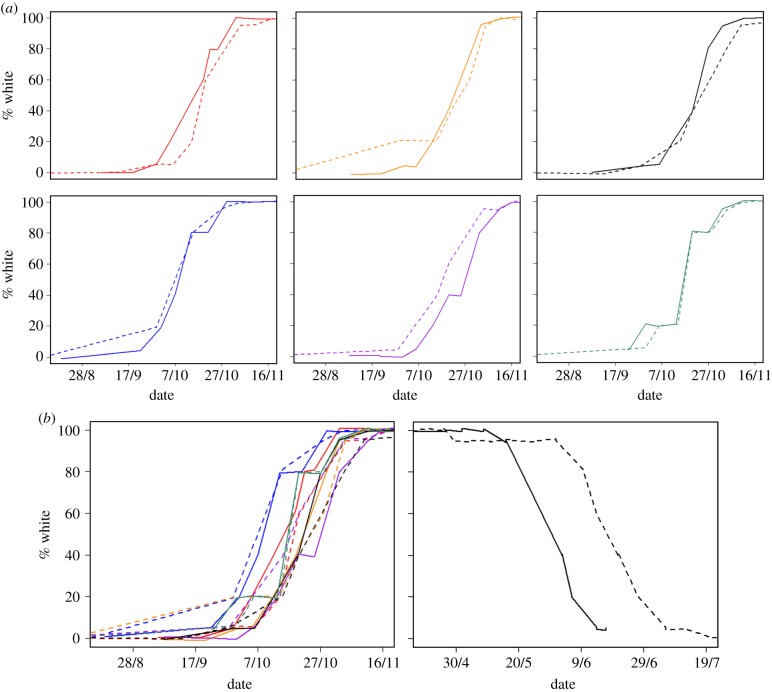
Spring moult phenology reaction norms of hares at the Gardiner study site, MT (17/9/2010 to 9/7/2012). (*a*) Autumn coat colour moult phenologies of six individual hares observed over two autumns. (*b*) Autumn (left) moult reaction norms of the individuals shown in (*a*) combined, and spring (right) moult reaction norm of one hare observed over two springs. Each coloured line represents reaction norms of a different individual in year 2011 (dashed line) and either 2010 or 2012 (full lines).

Finally, our limited data for the seven individuals that were observed over multiple seasons at the Gardiner site also indicated no plasticity in the autumn but some in the spring rate of moult. According to the plots, reaction norms of the six hares that we observed over two disparate autumns displayed similar phenologies, differing by only 0–10 days between the two autumns (figure 2*a*). By contrast, the one hare which was observed over two springs had moult phenologies that differed between springs by 15–20 days, comparable to the range of plasticity observed across all six individuals in the autumn moult (figure 2*b*).

### (b) Concealment

Contrary to our predictions, hares did not seek higher concealment with increasing colour contrast; rather, the level of concealment was mostly affected by season and site. First, we detected habituation to human observers, as concealment began to significantly decrease with number of location attempts when hares were located more than nine times. Therefore, we truncated the dataset to include only the first nine observations per hare which yielded a total of 731 observations from 139 radiocollared individuals at the two sites. Each individual was observed an average of 6.4 occasions (s.d. = 2.9).

The best model included season, site and quadratic form of colour contrast at 1 m (see the electronic supplementary material, S2, for the set of best models tested). The other three best models included one additional term each: snow at 10 m, whiteness, and snow at 1 m (in order) that had positive effects on concealment, but their 95% HPD intervals included zero. The significant quadratic relationship of contrast on concealment was in the opposite direction than expected, with highest concealment at medium levels of positive colour contrast and reduced concealment at high negative and high positive contrasts (*β*_Contrast_ = 0.0051, s.d. = 0.0021; *β*_Contrast_^2^ = −0.000085, s.d. = 0.000026; see the electronic supplementary materials, S3 and S4). Concealment varied seasonally, and hares were on average concealed by 25% more in the summer and 8% more in the autumn than in the winter, but not significantly different in the spring than in the winter. Concealment was significantly different at the two sites; hares at the Seeley Lake site were on average 11% more concealed than at the Gardiner site.

### (c) Flight initiation distance

Hares did not flee at farther distances with increasing mismatch as we predicted, but rather their concealment level, season and site variables played important roles in predicting their FID. We detected habituation to human observers, as FID began to significantly decrease with number of location attempts when hares were located more than five times. Therefore, we truncated the dataset to include only the first five observations per hare which yielded a total of 367 observations from 104 radiocollared individuals. Each individual was located on average 4.1 occasions (s.d. = 1.3).

Our set of best models included concealment, site, season, sex and either contrast or mismatch (see the electronic supplementary material, S5, for the set of best models tested). The model results can be interpreted as the increase in probability of flight initiation throughout the distance within which we measured the FID response (=20 m). Our first, second and sixth best model included mismatch at 10 m. Hares matched with their background were 5.6 times more likely to flee than brown hares on snowy background (hazard ratio_Negative Mismatch_ = 5.62, s.e. = 1.72), but there was no difference for white hares on brown background (hazard ratio_Positive Mismatch_ = 1.62, s.e. = 1.39; see the electronic supplementary material, S6, for a list of coefficients from the best model). A similar trend was observed in the fourth and fifth best model that included absolute mismatch at 10 m scale, where matched hares were about two times (fourth best model: hazard ratio_Abs Mismatch_ = 2.02, s.e. = 1.33; fifth best model: hazard ratio_Abs Mismatch_ = 1.87, s.e. = 1.33) more likely to flee than mismatched hares. The third best model included a linear relationship with colour contrast at 1 m. Hares were 0.77% more likely to flee with each 1% decrement in colour contrast (hazard ratio_Contrast_ = 1.01, s.e. = 1.00).

Concealment significantly decreased flight distance; with each 25% increment in body concealed hares were 26% less likely to flee. There was a significant difference in FID at the two sites; hares at the Gardiner site were 89% more likely to flee than hares at the Seeley Lake site. Season appeared in all best models but only summer was significantly different from winter; hares were 21% more likely to flee in summer than in winter. Sex was present in all best models as it improved model fit but was not significant in any of them. Similarly, snow at 1 m around hares and whiteness were present in three models of the best models set but their effects were not significant in any. According to the score tests and scatterplots of scaled Schoenfeld residuals, there was no evidence of non-proportional hazards in any of the terms in the best models.

### (d) Resting spots

**Figure 3.**
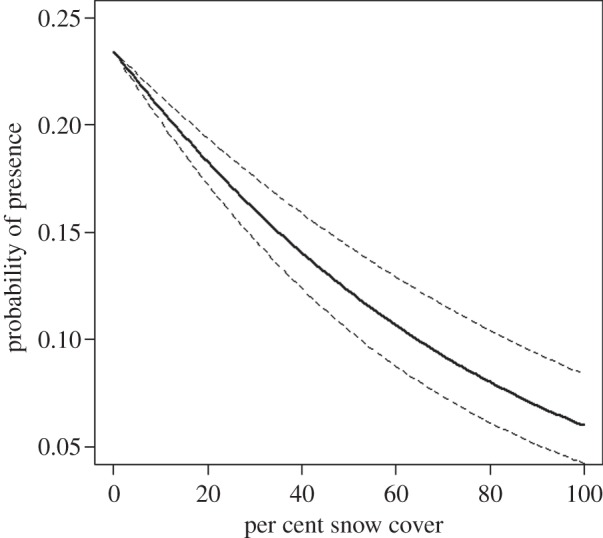
Probability of presence of a hare at a resting spot with percentage of snow cover at the Gardiner and Seeley Lake study sites, MT (9/17/2009 to 7/9/2012)*.* Dashed lines show 95% confidence intervals.

Hares were not more likely to rest at spots within their immediate vicinity where colour contrast was reduced but instead preferred spots with relatively little snow. Colour contrast was an important predictor of presence but hares were located at spots that resulted in higher colour contrast relative to the available spots within 10-m radius of the hares (*β* = 0.0076, s.d. = 0.0022). Further, we found strong evidence that hares were more likely to be found at spots with less snow cover. The probability that a hare would be found at a spot with no snow cover was 3.92 times higher than at a spot with complete (100%) snow cover (*β* = −0.016, s.d. = 0.0019, figure 3). The model which included snow cover received more support than the model including colour contrast (ΔAIC*_c_* = 63) or the null model (ΔAIC*_c_* = 72).

## 4. Discussion

Across a wide range of snow conditions and two study sites, snowshoe hares demonstrated little plasticity for modifying coat colour moult phenology or behaviours to track seasonal snowpack. The fixed initiation dates of coat colour moults are consistent with a photoperiod modulator of timing, as occurs for other circannual processes [33–35]. We observed no plasticity in the rate of the autumn brown-to-white moult on both the population (figure 1) and individual level (figure 2). Consistent with the findings of [13], we found plasticity in the rate of the spring white-to-brown moult with mean completion dates shifted by 19 days across two years of different snowpack. Additionally, we observed plasticity in the individual rate of moult in the spring. The one hare which was observed over two springs at the Gardiner site displayed different moult phenology each year, with a difference in spring moult rates nearly as large as observed across all of the six individuals over multiple autumn moults (figure 2).

The mechanisms for a fixed autumn moult phenology and only limited plasticity in the spring rate are unclear. One explanation for plasticity in the spring moult could be an elevated predation rate in the spring [36], placing higher selection pressure to adjust the rate of the spring moult as a means of optimizing camouflage against immediate snow conditions. Further, hares might simply be able to trace the change in the snow conditions better in the spring than in the autumn. Over three years and two study sites, we observed four substantial snow fluctuations (more than 30% and in the opposite direction of the seasonal change) in the weekly average snow cover around hares in the autumns and only one such fluctuation in the springs. Thus, the spring change in the snow conditions might be more predictable compared with autumn snow change, where early snowfalls are often followed by full melt-out before continuous winter snow cover builds up.

We did not detect any strong variables influencing the rate of the spring white-to-brown moult. Despite the more consistent snow change in the spring, our change point analysis indicated that snow cover explained only about a three-day shift in the average spring completion date. Temperature also was not a strong regulator of the spring moult rate as it only explained a one-day shift in the completion date. Finally, the rate of moult in the spring was not significantly influenced by sex, with females completing the spring moult on average two days earlier than males. The faster colour moult for females is consistent with previous observations [13,37,38].

The spring and autumn moults across the different study sites were similar in duration, each lasting about 40 days. Despite this similarity in moult length and limited coat colour plasticity within sites, natural selection appears to have aligned the moult phenology to correspond to average local climate at each site. Although the Gardiner site is slightly south of the Seeley Lake site, and therefore has a similar or slightly longer photoperiod, the higher elevation of the Gardiner site leads to considerably longer lasting snowpack. As might be expected with the longer snowpack, hares in the higher elevation Gardiner site obtained their white coats sooner in the autumn and retained them longer in the spring. Elevational and latitudinal gradients have been shown to affect the timing of the moults in several leporid species. Watson [15] showed that mountain hares (*Lepus timidus*) occupying high elevations became white earlier, turned dark later, and became whiter in winter than those at low elevations. Similarly, latitudinal differences were observed in museum specimens [39], where snowshoe hares from northern latitudes moulted from brown to white earlier in the autumn and retained the white coat longer.

We found that hares did not modify their hiding behaviour in a manner that reduced colour contrast at either the 1- or 10-m radius scale. If our assessment of the range at which predators visually perceive hares is correct, then hares mismatched to their surroundings within a radius of up to 10 m (an area of 314 m^2^) are more vulnerable to detection. The relationship between concealment and colour contrast suggested that hares most concealed themselves by vegetation when their coats were about 40% whiter than their immediate (1-m radius) background (40% contrast) and least when brown hares were on snowy backgrounds (−100% contrast; see the electronic supplementary material, S3). Because the effect size was small, this relationship may represent weak biological importance. Overall, concealment levels of hares seemed to be most affected by season and site, which may be good proxies for available vegetation cover. Hares were more concealed in the summer and autumn when leafy vegetation in the understory provided more horizontal cover than in the winter and spring when leafy vegetation was either absent or covered by snow.

Second, we did not find evidence for hares responding to colour contrast at either the 1- or 10-m radius scale by fleeing at a farther distance from a potential threat. FID of positively mismatched hares (white hares on brown background) was no different than for matched hares; negatively mismatched hares (brown hares on snowy background) consistently had the shortest FID. On the contrary, concealment had a strong effect on FID; hares that were most concealed stayed still longest. Shorter FIDs when concealment was high likely represented decreased perceptions of risk, as has been reported, for example, for pygmy rabbits (*Brachylagus idahoensis*; [22]). Seasonally, hares fled at longer distances in the summer than in the winter, consistent with our field observations that hares seemed to be more active in the summer (see also [40], who observed lowest activity for hares in winter). Longer flight distance in summer is also consistent with the general expectation that prey flee at farther distances when in better body condition [21]. Indeed, our personal field observations of hares during winter are that they generally appear calmer when approached, flee less even if they are fully exposed and often have their eyes closed, perhaps a strategy to save energy during winter periods of food limitation. Additionally, hares might perceive less predation risk in winter as their low foot loading provides a likely escape advantage from potential carnivores on snow [41].

Lastly, we found no evidence of hares preferring resting spots with colour background that would reduce colour contrast. On the contrary, hares were more likely to be found at spots within their immediate surroundings that increased their colour contrast with the background within 10-m radius. This is likely result of their overall preference to rest at spots with little snow cover. We noted this behaviour during field observations; when snow cover was heterogeneous on the landscape, hares were located at non-snowy spots such as under logs or in tree wells. Thermoregulation may therefore be playing an important role in resting spot choice.

Finally, we note several caveats and assumptions. First, our choices of 1- and 10-m radius of snow cover around hares might not represent the spatial scale of perceived colour contrast for hares and/or their predators. We did not consider larger areas than 10-m radius for estimating colour contrast based on the assumption that visual detection of hares by predators at such scales was unlikely in these relatively dense forests. Second, because little is known about the drivers of the circannual rhythm of the moult, our measurements of moult regulators (e.g. snow cover, cumulative sum of temperature degree days) may not capture the correct regulators of colour moult phenology in the wild. Likewise, hares may be pursuing behavioural strategies to reduce colour contrast that we did not measure. Hares and many of their predators are primarily crepuscular and nocturnal, and thus hares could be deploying anti-predatory strategies that minimize colour contrast during the main periods of activity. For example, hares could be foraging during the night preferentially at areas that match their coat colour or they could be modifying their FID in response to colour contrast during those times of the day. Finally, our use of human observers as a proxy for predators assumes that FID response to humans is the same as to hares’ natural terrestrial predators.

Nevertheless, across multiple years, two study sites, a wide range of snow conditions, and nearly 200 hares monitored in the wild, we find no evidence that hares perceive coat colour mismatch and act to shift hiding or flight behaviours or immediate microsite choice. We also confirm that moult initiation dates are fixed across years of different snowpacks, even as the moult phenology is locally shaped within regions by a combination of photoperiod and climate. Given the prospect for substantially decreased snowpack duration in the future due to climate change [13,42], it seems that the most likely avenue for reducing camouflage mismatch or its potential predation consequences in local populations is evolutionary shifts in moult phenologies, anti-predatory behaviours or in phenotypic plasticity.

## Notes

All handling procedures were approved by the University of Montana Animal Care and Use Committee (protocol no. 021-10SMWB-051110).

## Data accessibility

Data used in this paper are archived at the following website: http://workthatmatters.ncsu.edu/camouflage/publications/.
